# The Birth of a Child with Disability. Coping by Parents and Siblings

**DOI:** 10.1100/tsw.2003.63

**Published:** 2003-08-20

**Authors:** Isack Kandel, Joav Merrick

**Affiliations:** Faculty of Social Science, Department of Behavioral Sciences, Academic College of Judea and Samaria, Ariel, DN Ephraim 44837, Israel; National Institute of Child Health and Human Development, Ministry of Labour and Social Affairs, Jerusalem, Israel

**Keywords:** child health, disability, handicap, intellectual disability, developmental disability, mental retardation, parental coping, Israel

## Abstract

When a child is born, the life of the family changes significantly and each of its members must adapt to the new situation. When the child is born with a disability, in addition to regular adaptation, the family must cope with stress, grief, disappointments, and challenges, which may lead to a serious crisis or even disruption of family life.Parents must coordinate assessments, evaluations, and various treatments while maintaining contact with many professionals and numerous institutions or services. They find themselves faced with important decisions on behalf of the child, decisions on management of the child with disability, and economic decisions that will affect the whole family.This paper reviews the literature on the topic of coping when a child with disability is born and also studies the question as to whether a connection exists between parental orientation toward feelings of guilt and the family relationships system. The event of a child born with a disability is always a tragedy for the family, but early intervention and support may help the family to adjust and become positively involved in the care and development of the child, even if that child is different and in need of special treatment.

## INTRODUCTION

When a child is born, the life of the family changes significantly and each of its members must adapt to the new situation. When the child is born with a disability, in addition to regular adaptation, the family must cope with stress, grief, disappointments, and challenges, which may lead to a serious crisis or even disruption of family life.

Parents must coordinate assessments, evaluations, and various treatments while maintaining contact with many professionals and numerous institutions or services. They find themselves faced with important decisions on behalf of the child, decisions on management of this new situation, and economic decisions that will affect the whole family.

This paper reviews the literature on the topic of coping when a child with disability is born and also studies the question as to whether a connection exists between parental orientation toward feelings of guilt and the family relationships system.

## METHODS

In order to review the literature on coping, parental and sibling response to the birth of a child with disabilities, or a sibling, we conducted computerized searches using the JDC-Brookdale Institute Library in Jerusalem and our personal libraries. The computer search was conducted on Medline/PubMed/Medline Plus, Social Science Information Gateway (SOSIG), Scirus, APA on-line, PsychCrawler, Ingenta, World Wide Web Resources for Social Workers, Google, Altavista, and Infotrieve**.** Searches were conducted for “birth and handicapped child/disabled child/impaired child” and “sibling and handicapped/disabled/impaired child”. Part of the material was used for this paper.

## THE CHILD

Several researchers have found that children play a very important role in satisfying the needs of their parents[1,2]. Children can be associated with materialism or competition and their parents' aspirations to achieve certain status or their desire to expand the family tree. Therefore, it can be a terrible blow to the parents when their child is unable to fulfill these wishes.

In general, one can observe several areas in which giving birth to a child is important for the parents. The child may be seen as the physical and psychological extension of his/her parents, possessing the hereditary combination of the characteristics of the latter. Where the child displays “good” characteristics, it is interpreted as a reflection of the positive side of the parents. Likewise, if his/her individual characteristics are negative or abnormal, they can be interpreted as a concealed or overt reflection of the characteristics of the parents. In many societies children are almost universally seen as an extension of their parents.

The child can also be seen as a means to satisfy the wishes of his/her parents, i.e., where their wishes and desires can come true. However, the child is not always able to fulfill and realize those wishes. The child may also be seen as a way for parents to achieve “immortality”, by perpetuating their good name into the next generation. In some families the child is seen by the mother as a gift from her to her husband[3].

The disability of a child also makes a difference. Researchers from Montreal[4] studied groups of children and their parents: a group of children with Down syndrome, a group with congenital heart disease, a group with cleft lip and/ or palate, and a control group without disability. The study revealed that the parents of children with Down syndrome and those with congenital heart disease showed greater levels of parental stress and psychological distress in comparison to the other two groups. Mothers were found to report greater levels of stress and distress overall.

Later in life, the disabled child him/herself will have to cope with a variety of stress factors. Deutsch[5] describes at least three categories: stress factors experienced by everyone (for example, a death of a relative), factors that are not at all stressful to the general population (for example, going to a store), and stress factors that are unique to persons with a disability (such as not being able to handle money). In order to cope and preserve self-esteem, the child with a disability may create a private world of his/her own and use various defense mechanisms to survive emotionally.

## PARENTAL REACTION TO THE BIRTH OF A CHILD WITH DISABILITY

The birth of a child with a disability can cause disappointment to the parents[1] and the reaction of the families seem to follow the five stages of the Kubler-Ross grief elaboration theory (denial, anger, bargaining, depression, and acceptance)[6,7]. This reaction is similar to what we observe in parents with perinatal death or loss[8].

It should be emphasized that the functional crisis experienced by mothers and fathers of children with a disability may be accompanied by psychological stress, a feeling of loss, and low self-esteem. In addition, the fact that the child is unable to fulfill their expectations may also be disappointing. The birth of a child with a disability may result in a severe blow to the self-esteem of the parents, create disappointment, and result in the child becoming a social obstacle that will also cause feelings of shame and embarrassment.

Parental reactions to the diagnosis of the disability will not be identical. The intensiveness of reactions and their character depend on several dynamic factors, such as individuality, the character of social relations, feelings about the deviation, and the social status. In the literature[9], a wide range of reactions are mentioned, some considered more frequent than the others: anger, disappointment, shame, frustration, and grief. The coping process is not static, but a constantly changing cognitive and behavioral effort by the person to manage both external and also internal stress factors and pressures.

Anger, disappointment, and shame[6,7,8] — these reactions result from the fact that the child is not the ideal child that the parent anticipated. The child is unable to fulfill the hopes and ambitions he/she was expected to. There are cases when parents unconsciously consider the child to be responsible for crushing their ambitions (as if he/she is "deliberately" disabled). However, since many parents consider it inappropriate to direct their negative feelings toward the children, anger may also be directed towards the other parent or towards others (for instance, the physician or other professionals for a variety of reasons, such as having made an incorrect diagnosis, insensitivity, offering false hope, or providing inadequate or ineffective treatment or services). These feelings of jealousy and anger are common in many families. The emotions may also be directed towards other families who do not have to contend with such stress or those with disabled children who are higher functioning or whose children have improved to a greater extent. Sometimes, the opposite reaction can be observed, which is expressed in overprotecting the child. In other cases, the parents see the disabled child as a symbol of their own personal failure. The feelings of a damaged self-esteem give rise to intensive feelings of inferiority and shame.

Frustration[10] over the fact that the child is not able to fulfill the parents' expectations can become even deeper as the slow development of the child makes him/her totally dependent on the parents, often especially the mother, seriously limiting her independence and freedom. In addition, social and economic aspects of raising a child with disabilities may provoke additional anger and frustration (e.g., difficulties in maintaining social communication, leisure activities, work projects, or economic plans). Frustration can belong to one of two types: (1) frustration resulting from role organization factors, i.e., failure to organize a new role system, since the disabled child is not able to play the role he/she is expected to or (2) frustration resulting from the destruction of ambitions and wishes for a happy family life.

Often the initial diagnosis of the child's disability will produce a grief reaction in parents and other family members[3]. This may be the result of initial confusion and uncertainty. Grief and bereavement are normal reactions to the loss of an object (in this specific case the object is symbolic). By means of these feelings, the human being temporarily retreats from involvement in the external world and allows his/her ego to focus on transferring the mental energy from the object on which it was concentrated to an alternative object. Transferring the energy is essential for successful conclusion of the bereavement process. However, this solution is not possible in case of the birth of a disabled child, since there is no final separation from the lost object, the child exists; thus, there is no opportunity to grieve over him/her without experiencing constant demands from his/her side. As a result of the demands, which are opposite to releasing oneself from the lost object and accepting the child, a situation is created in which “chronic bereavement” is opposed to counter-feelings. Since this ambivalence cannot be accepted in the parents' consciousness, it is pushed aside and causes additional difficulties in finishing the lamentation process. Ambivalence towards the object is not a part of the usual bereavement, since the grief process itself is a temporary phenomenon.

Many parents also have little understanding of what the diagnosis of a given disease or syndrome entails and many will have various perceptions and speculations about the disease causing the disability. Parents should therefore be informed regarding the varied manifestations and aspects of the disability. Sometimes it is also very hard to predict the cause or development of the disability at an early age, which makes it even more difficult for the parents.

Grief is a complex reaction with the loss of the expected normal child and the necessity to develop a new role of attachment to the abnormal child. Olshansky[11] describes grief for a disabled child as a lifelong “chronic sorrow” that may accompany the parents all their life, regardless of whether the child lives at home or is in placement[12]. Although the intensity varies from one to another, it seems that all parents experience grief. Olshansky[11] argued that this type of grief should not be interpreted as a neurotic reaction, but rather it should be seen as a normal and natural reaction to the crisis.

The crisis can take the following forms[1,12]:

- **The change crisis:** this crisis takes place immediately after the diagnosis of the disability and is a most difficult experience. The parents are full of expectation for the birth of a normal child, and when they are informed about the disability, all their dreams are ruined, causing the traumatic reactions. This crisis is not a reaction to the handicap itself; rather, it is a reaction to the sudden change of reality.
- **The ideological crisis:** the change crisis is comparatively short, however, after the parents have digested the news, they must confront this experience every day. This confrontation gives rise to strong emotional reactions, leading to an ideological crisis, which may last for a longer period of time. The parents are in a state of constant ambivalence. On the one hand, they feel that they have to love and protect their child, but on the other hand, social values cause them to feel discomfort, feelings of failure, and inability to accept the child as a “beloved” one. Such characteristic reactions as guilt, shame, overprotection, and grief appear at this stage.
- **The reality crisis:** this crisis is directly related to the objective difficult conditions of bringing up a child with disability. The parents face numerous difficulties that influence their ability to manage the problem. The first difficulty is financial, since expenses grow considerably compared to their previous situation or to that of other families. Many parents are disturbed by fears related to the influence the child has and will have on their lifestyle. Family members may stay in seclusion at home and avoid spending their time in the way they used to, before the child was born. Many parents express concern regarding the coming of a time when they will not be able to take care of the child themselves.

The stages mentioned above are not necessarily pure, since there can be overlap, but in order to assist and support parents, it is important to realize at what stage of the crisis they are.

The issue of gender differences in coping strategies has been studied by Sullivan[13] with 150 parents following the birth of a child with Down syndrome. Each parent was requested to answer a questionnaire (COPE inventory). It was found that females scored significantly higher than males in the areas of seeking instrumental and emotional support, in focusing on and venting emotions, and suppression of competing activities. An additional study was carried out with 75 parents of young children, which displayed the same results. Although gender differences were found, no value may be ascribed to these different coping strategies.

Working through grief in the families of children with any disability is an on-going process with periods of greater and lesser intensity to the grieving. This intensity may relate partly to developmental issues and events, such as birthdays or other rites of passage (e.g., Bar Mitzvahs, graduations, marriages) and may underscore how different the child is from his/her typical peers. Grief intensity may also relate to more personal, individual factors. Sometimes there is an alternation of hope and despair. Each new treatment or program for the child is often accompanied by an increase in optimism in the parents. If the new treatment or program is deemed unsuccessful, despair may follow, only to be replaced by hope once again, when a new plan is implemented.

## PARENTAL GUILT

Guilt is another common reaction to the diagnosis of a disability in a child. Sometimes that has been caused by the medical and professional community, who directly or inadvertently attributed a disease or condition in a child as parental failure[14], which later turned out to be based on a genetic disorder. The possible contribution of additional factors such as environmental toxins has also been discussed. Many parents wonder if they unwittingly did something to contribute to the disability in their child (such as exposure to X-rays, mercury from injections or dental fillings).

An adult may experience guilt as a result of ideas and feelings that are interpreted as forbidden or negative. The birth of a disabled child makes his/her parents feel disappointment, anger, and hostility towards the child. These feelings are interpreted as negative and can arouse guilt feelings and unconscious expectation that child disappear. Guilty feelings are one of the most frequent reactions of the parents to the birth of a child with disability. There are parents who feel rejection, disappointment, and anger because their child is not the one they looked forward to. Since the parents cannot tolerate or suffer these negative feelings, they deny the feelings by directing the anger towards themselves — the feeling that this is a punishment for their past sins[12]. In other cases, the guilt feeling is directed (as a result of negative feelings) towards other people, such as a spouse or a physician, or towards spiritual matters. We have often heard the following statements by parents with disabled children: “This happened as a punishment for me leaving the religion;” “It is all my fault. Before I got married, I had been ‘flirting about’. I made love before marriage and I always felt terrible about that, and now I have to pay for it.”

Rosenzweig[15] has classified reactions to frustration by the following categories:

- Interpunitive reaction — a reaction of anger and guilt directed towards the “self”. This reaction takes place when the super-ego does not allow realization of the aggressive feelings; rather, it turns them into guilt feelings and regret by means of keeping a distance and isolation.

- Extrapunitive reaction — a reaction of anger and hostility towards the external world, considering other people to be guilty. In this case as the super-ego is weak, it allows expressing the hostility feelings.
- Impunitive reaction — reactions of shame and guilt are slight, there is a compromise with the problem. This reaction is possible when the ego is strong and the self-esteem is positive. In pathological cases there is a process of keeping a distance, but in normal cases there is a process of sublimation.

Sometimes the guilt and shame is related to the inability of the parents to communicate with each other or with other family members[12]. In Israel, we have seen over several years that parents leave their disabled children in the hospital. A nationwide study was conducted[16] during 1979–1983 and 1987–1991 to examine the factors affecting parental abandonment of infants with Down syndrome. The overall abandonment rate was 25%, where the major factors were the age of the mother, birth order, the health status of the child, and the study period. The trend to abandon these children has gradually been reduced over time.

## INFLUENCE ON PARENTAL MARRIED LIFE

The effects of the birth of a child with disabilities on the marriage of his or her parents have been studied by several researchers.

An early study (1964) conducted in Central California by Fowle at the University of the Pacific included a total population of 328 families child with severely intellectually disabled children aged 3–17 years. A selected experimental group of 35 families from a total group of 83 families who had hospitalized their child was matched with a control group of 35 families from a group of total 245 families who had not hospitalized their child. She could not find any significant difference in marital integration between the two groups, but there was a significant difference in the role tension of the siblings, especially in that of the oldest female sibling of the family[17].

A study of 142 families with a child born with spina bifida[18] (56 families with a surviving child) between 1964–1966 was examined in 1976. The divorce rate for families with a surviving child was found to be nine times higher than that for the local population and three times higher than for the families where the child with spina bifida had died. Marriages that followed a prenuptial conception were especially vulnerable with a separation/divorce rate of 50%. All divorced fathers and only one mother had remarried at follow-up. An additional study[19] also found the divorce rate ten times larger in the families with a disabled child than in the general population.

The disability can cause damage to the married life of the parents in several different ways: it can create strong parental feelings, it can be a depressing symbol of a common failure, it can change the family organization, or create fertile ground for conflicts[20]. One frequent problem is the fact that the burden for childcare is not divided equally between the parents. In the common situation, the father is generally at work, while the mother cares for the child with the disability. The parents must organize a system of roles and a division of the burden of work in order to prevent the burning out of one partner[21]. In addition, devotion of the mother to care for her child may make the father feel neglected, which sometimes can result in violence[22]. Sometimes the core of the conflict stems from the fact that each parent conceives the situation in a different way. One parent may relate to the child as a failed case, while the other as a capable, or even a normal child. In addition there are parents who are unable to live with what they see as shame or stigma[19].

It is therefore important that as soon as a child is born with a disability, the parents should have the opportunity to talk and discuss the various issues with a competent professional so that many adverse reactions can be prevented. This way the family can be helped to adjust and become realistically involved with the care and development of the child[23].

## INFLUENCE ON SIBLINGS

In the study from California[17] mentioned above, where two groups were compared (child with disability hospitalized vs. child at home), the sibling role tension was also investigated. When the child with disability was kept at home, 48 siblings in each group showed a significant difference with a higher sibling role tension in the group. The study also showed that the oldest female sibling in families with a disabled child at home displayed more role tension.

A study was conducted with 327 siblings of disabled children compared with 248 siblings from a random sample of families in order to examine if the early family environment of siblings of disabled children had an influence on psychological functioning[24]. The results showed that younger male siblings, and especially those in close age-spacing relationship to the disabled child, scored higher on the psychological impairment than older male siblings. Contrary to these findings, the study showed that younger female siblings were psychologically better off than older female siblings and their age-spacing was not significantly related to psychological functioning.

A study from the U.K. with 183 children with intellectual disability (95 with Down syndrome) and their nearest in age sibling (with classroom control of the sibling) showed that behavior problems in the sibling was found most often where the child with intellectual disability had disturbed behavior and especially in the Down syndrome group[25]. The siblings in the non-Down group showed more reading problems and behavior disturbance in school than either the Down and control group.

Results from a study at the University of Washington[26] of 110 children (8–15 years), half of whom had a disabled younger sibling, showed that distinct psychological predictors existed for the group with a disabled sibling with parental stress and some dimensions of the family social environment the most significant factors.

A 4-year follow-up study among orthodox Jewish families (82 families) in Illinois showed a decrease in the negative impact of the child and an increase in sibling and overall family adjustment. Parents cited religion as an important source of strength, while lack of time, behavior problems, and limited availability or use of professional assistance was a continuing difficulty[27].

One study looked at the attitude of the sibling towards their brother or sister with a disability[28] and found that some of the very young siblings wanted to be similar and tried to imitate their disabled sibling, especially if the sibling had a physical disability. Before the age of 2 years, children were able to recognize that their brother or sister were different and often imitated the parent's behavior towards the older child.

Researchers in Canada[29] studied unaffected siblings (137 in total) of children with pervasive developmental disorder (PDD) and Down syndrome with a control group over a 3-year period. A significantly greater number of adjustment problems were found in the siblings of PDD children and also caregivers of PDD reported the highest level of distress and depression, which persisted over the study period.

A recent Dutch study[30] looked at siblings of children with a physical disability (43 children) in order to investigate the sibling relationship, relationship with parents, and with others. The siblings reported difficulties with common activities and communication with the disabled sibling and expressed concern for the future and the health of their disabled sibling. Open communication and trust were the main characteristics in their relationship with parents, while having a sibling with a disability did not affect their relationship with friends.

In Israel, we have seen several cases in residential care for persons with intellectual disability where the parents kept it a secret from their family that they had a child with intellectual disability. The parents visited their child in the center, but the siblings knew nothing. When the parents died, the siblings became guardian without knowing that they had a sibling with a disability. In the last 10 years, therefore, we have made a major effort to involve the whole family in visiting the disabled child in residential care and support siblings who did not know of their brother or sister in care.

## OUT OF HOME PLACEMENT

Many parents find it hard to have their disabled child at home when they and the child grow older[31], especially when further health problems occur[32]. If the social welfare system in the country provides suitable care for ageing persons with intellectual disability at home, the person with a disability will stay longer at home or even in independent living. Sometimes the functional and health condition decline in the disabled child and that will force the parents to accept or seek out of home placement.

Families with high levels of internal resources were more likely to plan for the child to continue living at home[33], but the out of home placement is an on-going process and once the idea of placement is entertained, it still takes several years to act on[34].

The School of Education at the University of California[35] looked at risk factors for out of home placements in children with severe and profound intellectual disability. The study was a cross-sectional (n = 5,992) and two-panel (n = 141) design with predictor variables, such as age, sex, ethnicity, adaptive behavior, maladaptive behavior, level of intellectual disability, blindness, deafness, and epilepsy. It appeared from the study that factors such as age, adaptive behavior, and maladaptive behavior may be overstated and ethnicity played a larger role in placement.

A study from Australia[36] with a qualitative in-depth investigation of the everyday family life experiences of 167 families of young children with a disability showed that 125 (75%) definitely did not want to place their child, 32 (19%) were undecided, and 10 (6%) were actively seeking or had already sought placement. The factors that influenced placement were lack of congruence in everyday family life with regard to the child with special needs and the needs of other family members (family coherence), lack of integration of the child in everyday family life and the community (integration and quality future), and a concern about the effect of the child on the siblings now and in the future (sibling involvement and concern).

An interesting study performed in Aberdeen, U.K.[37] reported on a follow-up of a representative cohort of people with intellectual disability born between 1951 and 1955. At the age of 22 years, 60% (44 persons) were living at home with their parents and 40% in residential care (29 persons). At age 40 years, the most significant change was the expansion of the community sector, which accounted for half of the surviving cohort (24 out of 54 persons), 20 still living at home and 10 in hospital/long-term care. Out of home placements was associated with gender (male), challenging behavior, and the absence of one or both parents.

## CONCLUSIONS

This paper has looked on the effects on the family unit with the birth of a child with a disability. This event is always a tragedy for the family, but with early and sensitive care and intervention for the involved child, parents, and siblings, much can be done to help the family. This support can help the family to adjust and become positively involved in the care and development of the child, even if that child is different and in need of special care.

## BIOSKETCHES

**Isack Kandel, M.A., Ph.D.**, is senior lecturer at the Faculty of Social Sciences, Department of Behavioral Sciences, the Academic College of Judea and Samaria, Ariel, but during the period 1985–1993, he was director of the Division for Mental Retardation, Ministry of Social Affairs, Jerusalem, Israel. E-mail: Kandeli@aquanet.co.il

**Joav Merrick, M.D., D.M.Sc.,** is professor of child health and human development affiliated with the Zusman Child Development Center and Division of Community Health at the Ben Gurion University, Beer-Sheva, Israel and presently the medical director of the Division for Mental Retardation, Ministry of Social Affairs, Jerusalem and the director of the National Institute of Child Health and Human Development.

He has numerous publications in the field of child and human development, rehabilitation, intellectual disability, disability, health, welfare, abuse, advocacy, and prevention. Dr. Merrick received the Peter Sabroe Child Award for outstanding work on behalf of Danish Children in 1985 and the International LEGO-Prize (“The Children’s Nobel Prize”) for an extraordinary contribution towards improvement in child welfare and well being in 1987. E-mail: jmerrick@internet-zahav.net. Website: www.nichd-israel.com
